# Evaluation of human gene variant detection in amplicon pools by the GS-FLX parallel Pyrosequencer

**DOI:** 10.1186/1471-2164-9-464

**Published:** 2008-10-08

**Authors:** Roberta Bordoni, Raoul Bonnal, Ermanno Rizzi, Paola Carrera, Sara Benedetti, Laura Cremonesi, Stefania Stenirri, Alessio Colombo, Cristina Montrasio, Sara Bonalumi, Alberto Albertini, Luigi Rossi Bernardi, Maurizio Ferrari, Gianluca De Bellis

**Affiliations:** 1 Consiglio Nazionale delle Ricerche, Istituto di Tecnologie Biomediche (CNR-ITB), Via F. Cervi 93, I-20090 Segrate, Italy; 2 Genomic Unit for the Diagnosis of Human Pathologies, San Raffaele Scientific Institute, Milan, Italy; 3 Laboratory of Clinical Molecular Biology, Diagnostica e Ricerca San Raffaele S.p.A., Milan, Italy; 4 Vita-Salute San Raffaele University, Milan, Italy

## Abstract

**Background:**

A new priority in genome research is large-scale resequencing of genes to understand the molecular basis of hereditary disease and cancer. We assessed the ability of massively parallel pyrosequencing to identify sequence variants in pools. From a large collection of human PCR samples we selected 343 PCR products belonging to 16 disease genes and including a large spectrum of sequence variations previously identified by Sanger sequencing. The sequence variants included SNPs and small deletions and insertions (up to 44 bp), in homozygous or heterozygous state.

**Results:**

The DNA was combined in 4 pools containing from 27 to 164 amplicons and from 8,9 to 50,8 Kb to sequence for a total of 110 Kb. Pyrosequencing generated over 80 million base pairs of data. Blind searching for sequence variations with a specifically designed bioinformatics procedure identified 465 putative sequence variants, including 412 true variants, 53 false positives (in or adjacent to homopolymeric tracts), no false negatives. All known variants in positions covered with at least 30× depth were correctly recognized.

**Conclusion:**

Massively parallel pyrosequencing may be used to simplify and speed the search for DNA variations in PCR products. Our results encourage further studies to evaluate molecular diagnostics applications.

## Background

The availability of a reference human DNA sequence and high throughput technologies such as automated DNA sequencing has made the identification of sequence variations a key tool in several fields of modern biology. Resequencing of large sets of clinically relevant genes, in order to identify variants, is important for understanding the molecular basis of disease and, consequently, for developing diagnostic tests and identifying drug targets. Thus far, large resequencing projects have used a standard sequencing procedure in which gene fragments are amplified by PCR, purified and subjected individually to Sanger sequencing on both strands [1,2].

New-generation genome sequencing technologies have the potential to simplify this task. These new technologies are based on sequencing-by-hybridization [3], sequencing-by-ligation [4] or sequencing-by-synthesis [5,6]. The latter methodology, sequencing-by-synthesis, is implemented in the Genome Sequencer GS-FLX System (454 Life Sciences), which produces several hundred thousand DNA reads of at least 200 bp; this is done by monitoring the release of pyrophosphate during the growth of a DNA chain driven by a DNA polymerase [6]. The very high throughput of the instrument is achieved by massively parallel pyrosequencing reactions, which generate a highly redundant representation of the DNA regions under scrutiny.

The GS-FLX has been already employed in bacterial genome sequencing, miRNA discovery, cDNA sequencing, ultra-deep sequencing of PCR amplicons and in other fields of application . In a few papers, the technology has been used to generate detailed pictures of large genomic regions by either multiplexed PCR approaches [7,8] or direct genomic enrichment [9,10]. None of these studies, however, included a thorough analysis for known sequence variants. Therefore, in this study, we assessed the performance of massively parallel pyrosequencing in the blind, automated search for sequence variations within pools of PCR-amplified DNA from clinical samples.

## Results

We evaluated the performance of the new pyrosequencing technology of the GS-FLX (454 Life Sciences – Roche) in identifying sequence variants in pools of amplicons from human genomic DNA. We selected 16 genes associated with human genetic diseases (Table 1). Genes ranged in size from 4 to 50 exons and had marked allelic heterogeneity. We obtained PCR-amplified DNA corresponding to these genes from the DNA inventory of San Raffaele Hospital; all DNA samples had previously been sequenced by standard Sanger technique. Overall, 165 amplicons, containing 374 genetic variants, were obtained. These amplicons were mixed into four unequal pools (Supplementary Table 1 [see Additional file 2]) to test the ability of the sequencer to handle more or less complex DNA mixtures. Several amplicons were included in more than one pool, but always from different patients (not necessarily with the same sequences); therefore, we analyzed a total of 343 PCR products harboring 429 variants previously confirmed by Sanger sequencing: 350 heterozygous SNPs, 43 homozygous SNPs, 23 deletions from 1 to 44 bp (all but one in heterozygous state), and 13 heterozygous insertions from 1 to 7 bp.

**Table 1:** Reference information for 16 genes (165 amplicons, representing 374 different sequence variants) included in the study

Gene	OMIM	Reference sequence	Reference	Amplicons, n	Nucleotide variations, n
*ABCA3*	610921	NCBI-NM_001089	14	15	22

*ABCA4*	248200	NCBI-U88667	15	51	115
	604116				
	601718				
	153800				

*CACNA1A*	141500	ENSG00000141837	16	26	39
	108500				
	183086				

*CFTR*	219700	ENSG00000001626	17	13	22
	277180				

*EGR2*	607678	ENSG00000122877	*	4	6

*FTH*	134770	NCBI-NM_002032	18	5	10

*FTL*	600886	NCBI-NM_000146.3	19	5	12

*GJB1*	302800	ENSG00000169562	*	5	13

*HBB*	604131	NCBI-NT_009237	20	2	11

*IRP2*	147582	NCBI-NT_010194	*	5	16

*LAMIN A/C*	181350	ENSG00000160789	21	10	36
	159001				
	605588				
	115200				
	151660				
	248370				
	176670				

*MPZ*	118200	ENSG00000158887	*	5	14

*PMP22*	162500	ENSG00000109099	*	4	7
	118300				

*SFTPB*	267450	NCBI-M24461	14	4	10
	265120				

*SFTPC*	267450	NCBI-J03890	14	3	13
	610913				

*SLC40A1*	606069	NM_014585	23	8	28

* PCR primers and conditions available on request

Amplicon pools were sequenced following the standard GS-FLX procedure. The large set of reads generated was aligned to the reference genomic sequences, yielding a highly redundant representation of the target regions. Sequence variations were detected in blind, without knowledge of the previously determined genotypes.

### Amplicon pools and sequence coverage

The four pools contained from 27 to 164 of the 343 PCR products (Table 2 [see Additional file 1]). In each pool, equimolar amounts of each PCR product were used. Amplicons ranged from 121 to 569 bp (mean, 315 bp). The reference genome complexity ranged from nearly 9 Kb (pool 4) to over 50 Kb (pool 1), for a total of nearly 110 Kb to be resequenced. Sequencing with the GS-FLX generated over 373 000 reads for over 80.8 Mb of sequence. Blast mapping collected nearly 60 Mb (73%) of matched sequences; the remaining 27% was artifactual, mainly primer dimers, presumably generated during the original amplification reactions in which the amplicons were made. Mean read length was 222 bp, well within GS-FLX specifications. Pool 4 (the smallest) had a lower average read length (198 bp), possibly due to the over-representation of short amplicons. Overall, 104 700 bases (95,6%) of the reference sequence was covered at least 30× depth, considered the minimum necessary for reliable sequence variation detection based on preliminary experiments; 309 of the 343 amplicons were fully covered above this threshold. At a less restrictive 10× depth of coverage, 319 amplicons were fully covered (98,0% of bases).

### Identification of sequence variations

The redundant representation of input sequences was used to calculate the percentage base calls for every sequence position covered ≥ 30×. For positions with sequence heterogeneity, we considered only those in which the minor allele had an allelic fraction > 20%. Thus, 506 sequence variants were identified, with the smallest pool presenting 48 variants and the largest 221 variants (Table 3 [see Additional file 1]). The variants were classified, on the basis of the sequencing results from forward and reverse reads, into top confident (TC), very confident (VC) or not confident (NC) classes. Altogether, 41 variants were classified as NC and not further considered (none corresponded to sequence variations defined by Sanger sequencing). There were 379 TC calls, of which 357 (94,2%) were found to correspond to the known sequences once the data were unblinded; There were also 86 VC calls, of which 55 (64,0%) were correctly called. Thus, there were altogether 53 miscalls (TC and VC), giving a false-positive rate of 0,05% for the total 104 700 bases covered ≥ 30×. Considering the 393 SNP variations, the percentage agreement was 98,5% for TC calls (Supplementary Table 2 [see Additional file 2]). In contrast, for the 36 insertions and deletions, only 56,4% of TC and 28,6% of VC calls agreed with known sequences.

We also assessed the ability of the GS-FLX to identify the 429 sequence variations known by Sanger sequencing. Overall, 17 variants were missed: 15 heterozygous SNPs, 1 deletion and 1 insertion (Table 3 [see Additional file 1]). These 17 variants had all been covered at less than 30× depth, our cutoff for inclusion in the analysis. Had the coverage threshold been 10×, we would have identified seven of these missed variations, but we would have also included seven more miscalls, increasing the number of false positives (Supplementary Table 3 [see Additional file 2]).

**Figure 1:**
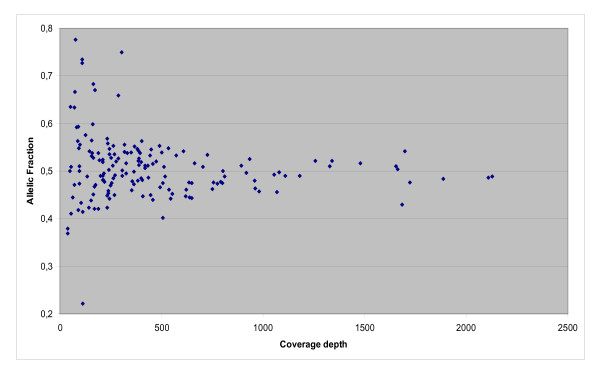
Relationship between sequence coverage with the GS-FLX and allelic fraction of heterozygous variations in pool 1.

In this study, we used a 30× sequence coverage as the minimum necessary for accurate calling with the GS-FLX. Actual coverage with this instrument exceeded 4000× in a few cases, and nearly 10% of the total 110 Kb sequence had an average coverage depth above 1000×. To understand the importance of coverage depth on the accuracy of the sequence calls, we plotted coverage vs. allelic fraction of the heterozygous variants identified in pool 1 (Figure 1). This analysis showed decreasing variability in allelic fraction with increasing coverage; as coverage increased, the values approached 0.5, the theoretical allelic fraction for one allele of a biallelic polymorphism.

### Impact of homopolymers and large indels

The 53 false positives (40 deletions, 7 insertions, and 6 heterozygous SNPs) obtained with pyrosequencing were checked by repeat Sanger sequencing, which in all cases confirmed the GS-FLX error. These miscalls were all observed to lie in homopolymers, i.e. stretches of at least three consecutive repeats of the same nucleotide. To understand the role of homopolymers in generating false-positive miscalls with the GS-FLX, we determined the presence of this sequence pattern in the 110 Kb DNA under scrutiny (Table 4 [see Additional file 1]. We identified 6840 homopolymers from 3 to 9 bp in length, for a total of over 23 Kb (21% of the DNA sequenced). Thus, only a very small fraction of homopolymers (53 of 6840; 0,78%) was associated with a miscall. However, the probability of a miscall increased with the length of the homopolymer; in particular the 108 homopolymers of 6 or more bp (1.6% of total) were associated with 26 (48,1%) of the 53 miscalls. In contrast, homopolymers were not associated with any false-negative calls: considering sequence positions covered ≥ 30×, a total of 132 sequence variants known by Sanger sequencing were contained within homopolymers (Supplementary Table 4 [see Additional file 2]). All were correctly identified.

Finally, since the Blast mapping procedure used in the study was found, in preliminary work, to fail on deletions or insertion longer than 10 bp, we devised a separate Blast procedure to analyze GS-FLX reads for these variants. This procedure found no large insertion but, in pool 4, one 44-bp heterozygous deletion in exon 49 of *Abca4*. These findings agree with the variants known by Sanger sequencing.

## Discussion

We assessed the potential of massively parallel pyrosequencing to identify sequence variants in pools of amplicons. Sequencing specificity at reasonable coverage depth (30×) was better than expected, with only 0,05% false-positive calls. Furthermore, the specificity of detection of SNPs was excellent (98,5% for TC calls) whereas errors were mostly related to indels, all lying within homopolymers. False negatives, of primary relevance in diagnostic applications, were absent, provided that 30× coverage depth was attained.

In order to widen the applicability of our approach, we purposely avoided any primer redesign or primer resynthesis (including primer tails for 454 sequencing as required by the GS-FLX protocol for amplicon sequencing). Tailed oligomers can help in sequencing an amplicon on one side. If both sides are to be sequenced (for increasing reliability and for covering amplicons larger than 250 bp as in this study) one should purchase two new primer couples and run two separate amplifications for each amplicon in the pool. In contrast, library preparation following the conventional 454 sequencing approach (Supplementary Figure 1) yields the required material with little additional time compared to the doubling of amplicon preparation and processing. Indeed, this approach could be of interest to all those who routinely identify sequence variations using any PCR-based technique.

The sample material in this study consisted of amplicons that varied greatly in length, sequence composition and sequence variations. Several of these amplicons were from the same genomic region amplified under the same conditions from different patients, with and without such mutations. We chose this experimental design in order to explore the performance of this approach depending on the molecular complexity under investigation. The pooled DNA samples were subjected to 454 sequencing and the resulting highly redundant representation of the targeted regions was used for blind, bioinformatics identification of sequence variants.

We attempted to normalize the concentrations of amplicons in the pools in order to minimize the variability in the coverage depth among different DNA fragments. However, despite accurate measurements of concentrations before pooling, there was substantial variability in the depth of coverage. Nonetheless, a very high average coverage was attained (400×). A relevant fraction of sequence positions (4%, corresponding to nearly 5 of 110 Kb) was below 30× coverage, thus preventing a reliable call according to our predefined parameters. With less stringent 10× coverage, 2 Kb was still below the threshold. On the other hand, nearly 10% of the sequence had an average coverage depth above 1000×, peaking at over 4000× in a few cases. These areas of great coverage "waste" a considerable proportion of sequencing power, making this approach less productive than expected and requiring the collection of many more reads than the minimum necessary to exhaustively cover the entire region under scrutiny.

The variability in amplicon coverage may depend on length and GC content, which affect amplification efficiency in emulsion PCR [4]. Alternatively, it may depend on ligation efficiency, which is affected by the sequence of the 3' and 5' ends [11]. We found no clear relationship between coverage and either amplicon length or GC content. However, similar problems have been observed in a large resequencing project using the Sanger method, despite the possibility of PCR and sequencing optimization [1]. In a defined diagnostic setting, notwithstanding the reason for such biased (variable) coverage, this problem could be solved by increasing the quantity of "low-yield" amplicons with respect to that of "high yield" ones [7]. After a few trials, normalization conditions could be defined and used for every new sample.

### Definition of parameters and identification of sequence variations

Sequence variations were searched for by comparing actual base calls to the expected sequence known from Sanger sequencing. In preliminary experiments, we evaluated data with 10× and 30× coverage depth, and observed that 10× coverage did not guarantee an allelic fraction (representation of the two alleles) of at least 20%. Therefore, we set our coverage threshold to 30×. To further improve the confidence of the sequencing results, we devised a classification system based on empirical observations. When both strands called the same variation, the call was classified as "top confident" (TC) call; considering only SNPs, only 5 of 340 TC calls were not confirmed; these miscalls were within homopolymers. When just one strand was available for sequence recognition (typically in amplicons longer that 250 bases when the variation was close to the amplicon's ends), the variation was classified as "very confident" (VC); only 1 of 44 VC SNP variants was a miscall, again lying within a homopolymer. When there were conflicting results between forward and reverse strands, we gave a "not confident" (NC) classification; this was the case in only 41 base positions. None of these NC calls corresponded to a true variation. Such miscalls were found in amplicons longer than 250 bp and were due to a decrease of sequence quality at the end of the read on one strand. Although this does not seem to be a general problem, when these miscalls occur, they can be simply discarded based on automated classification and visual inspection.

Pyrosequencing is known to be affected by over- or undercalling within homopolymers [12]. Although the combined length of homopolymers totaled nearly 23 Kb, we had just 53 false-positive calls (forty 1-bp deletions, seven 1-bp insertions, and six SNPs) lying within these regions. The miscall rate increased with homopolymer length, up to 9 bp in this study. In continuing experimentation, we have preliminary observations that, in longer homopolymers (e.g. 15- to 20-mers), the length of false-positive deletions also increases beyond single base pairs (unpublished observations). However, since the GS-FLX correctly called 132 sequence variations within homopolymers, these regions should not always be considered unreliable. Nonetheless, deletions and insertions in long homopolymers should be carefully evaluated.

Considering the four pools separately, no clear indication of a specific bias for accuracy was found. However, the smallest pool had the poorest performance in terms of mean coverage.

### Evaluation of diagnostic applicability

False positives (overcalling of variations) were reasonably low: we demonstrated an overall 99,95% specificity in base calling (53 miscalls within 104.7 Kb of sequence covered ≥ 30×). All these miscalls were in homopolymeric sequences and more frequently involved deletions and insertions (n = 47) rather than SNPs (n = 6).

False negatives are a major issue in diagnostics applications. In this study, they were absent among amplicons at ≥ 30× coverage (100% sensitivity). However, 17 of 429 known sequence variations (4,0%) were missed due to insufficient coverage. Decreasing the minimum coverage to 10× led to the identification of 7 additional variants. Nevertheless, with such a low coverage 7 additional miscalls were added, thus lowering specificity. In addition, a decrease in coverage depth was associated with a greater variability in allelic fraction, making it difficult to reliably call heterozygous variations. Indeed, high redundancy represents one of the major advantages of this approach, allowing for a detailed molecular description of complex mixtures of nucleic acids [13]. Therefore, according to our experience, a minimum 30× coverage depth is required for reliable detection of variants for diagnostic purposes.

The failure of pyrosequencing to adequately cover certain amplicons may necessitate that standard sequencing be performed on those DNA regions. However, as suggested earlier, in diagnostic laboratories that routinely sequence a defined set of amplicons, countermeasures can be adopted to improve the coverage of amplicons.

## Conclusion

This study confirms the high potential of massively parallel pyrosequencing in the scanning of DNA samples for sequence variations. Compared to traditional sequencing technology, this system is capable of higher throughput and is able to rapidly collect genomic information. Our study highlighted some critical aspects of the technology related to the uniformity of coverage. Based on our observation that the allelic fraction of variants approaches 0.5 as sequence coverage increases from 30× to over 2000×, in future work coverage depth should be carefully considered, in particular for diagnostic applications; this is a fundamental issue for the reliable detection of heterozygous variants. Moreover, since most sequencing errors were due to indels in homopolymeric tracts, analytical approaches might be specifically developed to better assess these variants. Our results encourage future studies evaluating the diagnostic applications of this sequencing technology in diseases with high allelic and genetic heterogeneity.

## Methods

### DNA samples

We selected 16 human genes associated with human genetic diseases (Table 1) and obtained corresponding PCR-amplified material from the clinical inventory of San Raffaele Hospital. A total of 343 amplicons were obtained, representing 429 genetic variations already identified by Sanger sequencing. DNA samples had been obtained from patients who had given informed consent or under protocols approved by the hospital's ethics review board.

For all amplicons in the study, PCR had been performed as previously described [14-22] or according to standard procedures (indicated by an asterisk in Table 1). Amplified regions comprised entire exons with intron-exon junctions or exon portions. Amplicons were checked for quality on agarose gel electrophoresis and directly quantified after ethidium bromide staining using a gel scanner (Typhoon 8600, Amersham).

### Amplicon pools

Amplicons were pooled into 4 samples in equimolar concentrations (Supplementary Table 1 [see Additional file 2]). On several occasions, one amplicon was included in two or more pools. PCR products bearing the same variation were from different individuals, in order to increase the variability under investigation.

Pools were purified using silica spin-columns (MinElute PCR purification kit, Qiagen, Valencia, CA). Each pool contained 2 μg DNA determined spectrophotometrically (NanoDrop Technologies, Wilmington, Delaware USA). The pools were checked for quality by capillary electrophoresis (Agilent Bioanalyzer 2100 with the DNA 1000 kit; Agilent Technologies, Palo Alto, CA, USA).

### GS-FLX pyrosequencing

Pools were prepared for FLX sequencing following the Low Molecular Weight DNA protocol as reported in the 454 Roche GS-FLX DNA Library Preparation Kit User Manual. Each pool was separately loaded onto one-fourth of a sequencing plate. Sequencing was performed according to the manufacturer's instructions.

### Sequence data analysis

GS-FLX reads were mapped to reference sequences using Blast v. 2.2.15 [23], with e value set to 1e-7; low complexity filter (DUST) disabled; word size, 4; and -v and -b set to the highest possible number of matches (in a dataset containing 100 000 reads, -v and -b are set to 100 000 or higher). Other parameters were left at default. References sequences were those of the NCBI human genome, build 36.3. The Blast output was stored in a PostgreSQL database together with the corresponding read and reference. Using each reference to collect its corresponding read set from the database, the resulting multiple alignment was used to calculate the percentage base call for every sequence position with a coverage depth of 30 (or 10), using a standard query language (SQL) query (available upon request). We defined a sequence variation when unexpected calls (with respect to the reference sequence) exceeded a 20% allelic fraction (allelic fraction = mutated allele counts/(wildtype allele counts + mutated allele counts)) in regions having 30× (or 10×) depth coverage. The detailed bioinformatics procedure will be published separately.

Since preliminary work revealed that this Blast procedure was inefficient on deletions and insertions larger than 10 bp, we further processed Blast outputs for reads that did not uniquely match to the reference genome, searching for those matching a single reference (amplicon) sequence twice with proper strandedness.

Sequence variants were automatically classified by an SQL query (available upon request) as follows:

- Top confident (TC): variation called by both forward and reverse reads;

- Very confident (VC): variation called in consensus regions where reads were available from one of the two strands only;

- Not confident (NC): conflicting calls between forward and reverse strands.

To visualize the sequence variants, the SeqMan package (DNASTAR, Madison, WI, USA) was used.

## Competing interests

The authors declare that they have no competing interests.

## Authors' contributions

The study was conceived and coordinated by AA, LRB, MF and GDB, and designed by PC, SBe, LC, MF and GDB. RaB devised the software procedure. RoB and ER generated the 454 sequencing data. SS, AC, CM and Sbo did the Sanger sequencing. Sequence alignment was done by RoB, RaB, ER, PC, SBe, LC, SS, AC, CM, SBo. The manuscript was written by RoB, RaB, ER, MF and GDB.

## Supplementary Material

Additional file 2 **Suppl. Fig 1, Suppl Table 1, Suppl Table 2, Suppl Table 3, Suppl Table 4**. **Supplementary Figure 1**. Schematics of the 454 sample processing and sequencing flow. **Supplementary Table 1**. Pool composition. **Supplementary Table 2**. Summary of variations. **Supplementary Table 3**. Comparison of results at 30× and 10× coverage. **Supplementary Table 4**. Summary of variations in homopolymers. Click here for file

Additional file 1 **Tables 2, 3,4**. **Table 2**. Composition of four amplicon pools and results of GS-FLX pyrosequencing. **Table 3**. Sequence variations identified by GS-FLX pyrosequencing and comparison to variants known from Sanger sequencing. TC, top confidence; VC, very confident; NC, not confident. **Table 4 **Homopolymers present in the 110 Kb DNA sequenced in this study, and relationship to false-positive sequencing calls. Click here for file
