# Supplementary material for: Evaluation of human gene variant detection in amplicon pools by the GS-FLX parallel Pyrosequencer
Source: BMC Genomics. 2008 Oct 8;9:464. doi: 10.1186/1471-2164-9-464 (PMC2569949; doi:10.1186/1471-2164-9-464)
Supplement: Additional file 2 — Suppl. Fig 1, Suppl Table 1, Suppl Table 2, Suppl Table 3, Suppl Table 4. Supplementary Figure 1. Schematics of the 454 sample processing and sequencing flow. Supplementary Table 1. Pool composition. Supplementary Table 2. Summary of variations. Supplementary Table 3. Comparison of results at 30× and 10× coverage. Supplementary Table 4. Summary of variations in homopolymers. [file 1471-2164-9-464-S2.doc]

Supplementary figure 1

Schematics of the 454 sample processing and sequencing flow

*Adaptor ligation*

*amplicon DNA pools*

**454 Sample preparation procedure**

**Single strand preparation and quantitation.**

**5’-Biotin moiety**

**A**

**B**

*Magnetic*

*streptavidin*

*coated*

*beads*

*Melting*

NaOH

**sstDNA Quantification**


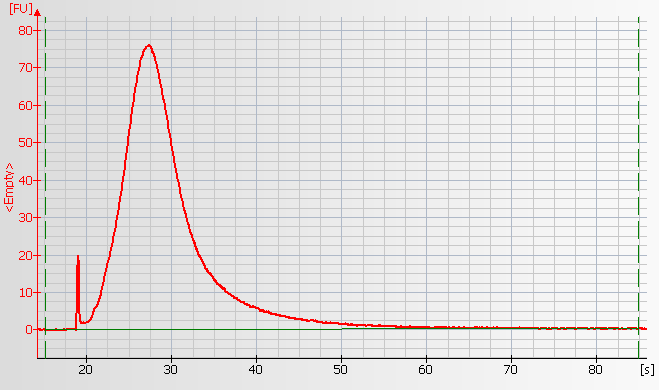


**sstDNA Recovery**

**No further processing**

*Library immobilisation*

*Amplification primer*

*sstDNA*

*capture Beads*

*Emulsion PCR*

*capture*

5’

3’

5’

3’

*Enrichment*

*Magnetic*

*streptavidin*

*coated*

*beads*

*NaOH Melting*

*Sequence*

*primer*

*annealing*

*Sequencing*

*primer*

*DNA beads*

*No further processing*

*5’-Biotin moiety*

*Null beads*

**Sample preparation : from emulsion PCR to PicoTiterPlate loading**

*PicotiterPlate loading and Sequencing*

*DNA beads Count*


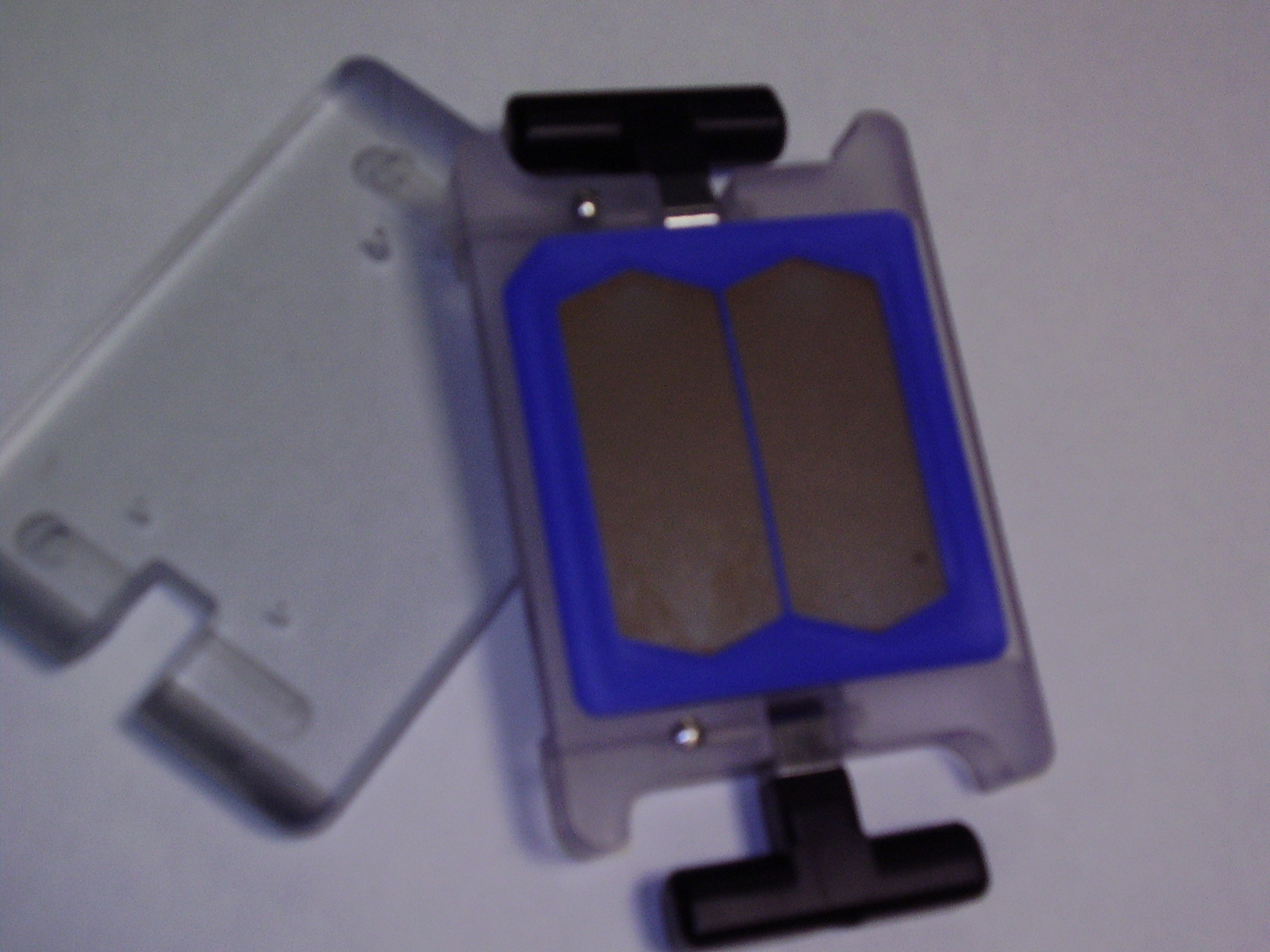


| Supplementary table 1 | | | Pool composition |  |  |  |
| --- | --- | --- | --- | --- | --- | --- |
|  |  |  |  |  |  |  |
|  |  |  | Pool | | | |
| Gene | Amplicon | Length | 1 | 2 | 3 | 4 |
| *ABCA3* | 3 | 221 | X |  |  |  |
|  | 4 | 491 | X | X |  |  |
|  | 5 | 351 | X | X | X | X |
|  | 6 | 394 | X | X |  |  |
|  | 7 | 477 | X | X |  |  |
|  | 12 | 393 | X |  |  |  |
|  | 13 | 368 | X |  |  |  |
|  | 14 | 372 | X | X |  |  |
|  | 20 | 450 | X | X |  |  |
|  | 22 | 504 | X |  |  |  |
|  | 24 | 317 | X |  |  |  |
|  | 25 | 350 | X |  |  |  |
|  | 26 | 275 | X |  |  |  |
|  | 27 | 473 | X | X |  |  |
|  | 28 | 426 | X |  |  |  |
| *ABCA4* | 1a | 382 | X |  |  |  |
|  | 1b | 405 | X |  |  |  |
|  | 2 | 289 | X |  |  |  |
|  | 3 | 301 | X | X | X | X |
|  | 4 | 258 | X |  |  |  |
|  | 5 | 279 | X | X |  |  |
|  | 6 | 287 | X | X | X | X |
|  | 7 | 204 | X |  |  |  |
|  | 8 | 398 | X | X |  |  |
|  | 9 | 272 | X |  |  |  |
|  | 10 | 280 | X |  |  |  |
|  | 11 | 324 | X | X |  |  |
|  | 12 | 362 | X | X | X |  |
|  | 13 | 277 | X | X | X |  |
|  | 14 | 406 | X | X | X | X |
|  | 15 | 405 | X | X |  |  |
|  | 16 | 309 | X | X |  |  |
|  | 17 | 232 | X | X |  |  |
|  | 18 | 280 | X |  |  |  |
|  | 19 | 322 | X | X | X |  |
|  | 20 | 270 | X |  |  |  |
|  | 21 | 248 | X | X |  |  |
|  | 22 | 334 | X | X | X | X |
|  | 23 | 384 | X | X |  |  |
|  | 24 | 212 | X | X |  |  |
|  | 25 | 379 | X |  |  |  |
|  | 26 | 246 | X |  |  |  |
|  | 27 | 349 | X | X |  |  |
|  | 28 | 291 | X | X | X |  |
|  | 29 | 263 | X | X | X |  |
|  | 30a | 232 | X | X |  |  |
|  | 30b | 213 | X | X |  |  |
|  | 31 | 229 | X |  |  |  |
|  | 32 | 239 | X | X | X |  |
|  | 33-34 | 501 | X | X | X |  |
|  | 35 | 336 | X | X |  |  |
|  | 36 | 346 | X | X |  |  |
|  | 37 | 260 | X |  |  |  |
|  | 38 | 302 | X | X |  |  |
|  | 39 | 274 | X | X |  |  |
|  | 40 | 285 | X | X | X |  |
|  | 41 | 299 | X | X |  |  |
|  | 42 | 213 | X | X |  |  |
|  | 43 | 298 | X |  |  |  |
|  | 44 | 287 | X |  |  |  |
|  | 45 | 257 | X |  |  |  |
|  | 46 | 257 | X | X |  |  |
|  | 47 | 258 | X |  |  |  |
|  | 48 | 353 | X | X |  |  |
|  | 49 | 220 | X | X | X | X |
|  | 50 | 448 | X | X |  |  |
| *HBB* | 1 | 147 | X | X | X | X |
|  | 2 | 415 | X | X | X | X |
| *CACNA1A* | 1a | 322 | X |  |  |  |
|  | 1b | 326 | X |  |  |  |
|  | 4 | 159 | X | X |  |  |
|  | 5 | 291 | X |  |  |  |
|  | 6 | 346 | X | X | X |  |
|  | 8 | 212 | X | X |  |  |
|  | 9 | 179 | X |  |  |  |
|  | 11 | 309 | X |  |  |  |
|  | 13 | 263 | X | X |  |  |
|  | 15 | 213 | X |  |  |  |
|  | 16 | 268 | X | X |  |  |
|  | 19b | 398 | X | X |  |  |
|  | 19c | 315 | X | X | X | X |
|  | 20a | 427 | X | X |  |  |
|  | 20b | 160 | X |  |  |  |
|  | 23 | 121 | X |  |  |  |
|  | 25 | 215 | X |  |  |  |
|  | 26 | 229 | X |  |  |  |
|  | 27 | 296 | X |  |  |  |
|  | 32 | 236 | X |  |  |  |
|  | 36 | 314 | X | X |  |  |
|  | 40 | 195 | X |  |  |  |
|  | 42 | 266 | X |  |  |  |
|  | 45 | 330 | X | X |  |  |
|  | 46 | 312 | X |  |  |  |
|  | 47 | 169 | X |  |  |  |
| *CFTR* | 2 | 194 | X |  |  |  |
|  | 3 | 258 | X |  |  |  |
|  | 4 | 437 | X | X | X |  |
|  | 5 | 196 | X | X | X |  |
|  | 7 | 390 | X | X |  |  |
|  | 11 | 197 | X | X | X | X |
|  | 12 | 379 | X |  |  |  |
|  | 13a | 528 | X |  |  |  |
|  | 14b | 175 | X |  |  |  |
|  | 17b | 379 | X |  |  |  |
|  | 19 | 455 | X | X | X |  |
|  | 20 | 393 | X | X |  |  |
| *EGR2* | 3 | 384 | X |  |  |  |
|  | 4 | 352 | X |  |  |  |
|  | 5 | 459 | X | X |  |  |
|  | 6 | 315 | X | X |  |  |
| *SLC40A1* | 1 | 571 | X | X | X | X |
|  | 2 | 277 | X | X | X |  |
|  | 4 | 328 | X | X | X |  |
|  | 5 | 283 | X | X | X | X |
|  | 6 | 337 | X | X | X | X |
|  | 7a | 483 | X |  |  |  |
|  | 7b | 414 |  | X | X |  |
|  | 8 | 409 | X | X |  |  |
| *GJB1* | P1 | 439 | X | X |  |  |
|  | P2 | 482 | X | X |  |  |
|  | 1 | 361 | X | X | X | X |
|  | 2 | 431 | X | X | X | X |
|  | 3 | 387 | X | X |  |  |
| *FTH* | IRE | 246 | X | X |  |  |
|  | 1 | 237 | X |  |  |  |
|  | 2 | 332 | X | X |  | X |
|  | 3 | 266 | X |  |  |  |
|  | 4 | 269 | X |  |  |  |
| *IRP2* | 5 | 363 | X | X |  |  |
|  | 7 | 305 | X | X | X |  |
|  | 10 | 305 | X | X |  |  |
|  | 14 | 222 | X | X | X |  |
|  | 21 | 269 | X | X |  |  |
| *FTL* | IRE | 359 | X | X |  |  |
|  | 1 | 278 | X |  |  |  |
|  | 2 | 323 | X | X |  |  |
|  | 3 | 357 | X |  |  |  |
|  | 4 | 287 | X |  |  |  |
| *LAMIN A/C* | 1A | 349 | X | X | X | X |
|  | 2 | 338 | X | X |  |  |
|  | 3 | 232 | X |  |  |  |
|  | 4 | 341 | X | X | X | X |
|  | 5 | 231 | X | X | X | X |
|  | 6 | 375 | X | X | X | X |
|  | 7 | 358 | X | X | X | X |
|  | 9 | 235 | X | X | X | X |
|  | 10 | 320 | X | X | X | X |
|  | 11 | 408 | X | X |  |  |
| *MPZ* | 1 | 293 | X | X |  |  |
|  | 2 | 274 | X | X | X |  |
|  | 3 | 336 | X | X | X | X |
|  | 4 | 269 | X | X | X |  |
|  | 5-6 | 452 | X | X | X | X |
| *PMP22* | 2 | 273 | X | X |  |  |
|  | 3 | 290 | X | X |  |  |
|  | 4 | 330 | X | X | X | X |
|  | 5 | 314 | X | X | X |  |
| *SFTPB* | 1 | 237 | X |  |  |  |
|  | 3 | 202 | X |  |  |  |
|  | 4 | 265 | X | X | X | X |
|  | 7 | 288 | X | X | X |  |
| *SFTPC* | 1 | 260 | X | X |  |  |
|  | 2 | 313 | X | X | X |  |
|  | 4 | 278 | X |  |  |  |

Supplementary table 2

|  | **Total variants** | | **SNPs** | | **Ins/dels** | |
| --- | --- | --- | --- | --- | --- | --- |
|  | Called | Confirmed (%) | Called | Confirmed (%) | Called | Confirmed (%) |
| TC | 379 | 357 (94%) | 340 | 335 (98%) | 39 | 22 (56%) |
| VC | 86 | 55 (64%) | 44 | 43 (98%) | 42 | 12 (29%) |
| NC | 41 | 0 | 1 | 0 | 40 | 0 |
| Total | 506 | 412 (81%) | 385 | 378 (98%) | 121 | 34 (28%) |

Supplementary table 3

| Coverage | Total Calls (TC+VC) | Confirmed Calls (TC+VC) | Miscalls (TC+VC) |
| --- | --- | --- | --- |
| 30X | 465 | 412 | 53 |
| 10X | 479 | 419 | 60 |
| Sanger | 429 |  |  |

Supplementary Table 4

| All Pools | Correct calls related to Homopolymers | | | |  |
| --- | --- | --- | --- | --- | --- |
| Homopolymer  Length | A | C | G | T | Total |
| 3 | 11 | 26 | 36 | 7 | 80 |
| 4 | 0 | 11 | 16 | 1 | 28 |
| 5 | 3 | 5 | 8 | 5 | 21 |
| 6 | 0 | 1 | 0 | 0 | 1 |
| 7 | 0 | 1 | 1 | 0 | 2 |
| 8 | 0 | 0 | 0 | 0 | 0 |
| 9 | 0 | 0 | 0 | 0 | 0 |
| Total | 14 | 44 | 61 | 13 | 132 |
