# Supplementary material for: Evaluation of human gene variant detection in amplicon pools by the GS-FLX parallel Pyrosequencer
Source: BMC Genomics. 2008 Oct 8;9:464. doi: 10.1186/1471-2164-9-464 (PMC2569949; doi:10.1186/1471-2164-9-464)
Supplement: Additional file 1 — Tables 2, 3,4. Table 2. Composition of four amplicon pools and results of GS-FLX pyrosequencing. Table 3. Sequence variations identified by GS-FLX pyrosequencing and comparison to variants known from Sanger sequencing. TC, top confidence; VC, very confident; NC, not confident. Table 4 Homopolymers present in the 110 Kb DNA sequenced in this study, and relationship to false-positive sequencing calls. [file 1471-2164-9-464-S1.doc]

**Table 2**

Description, reads coverage and sequencing results of pools

| Pool | N. of amplicons | Input  Sequence  (Kb) | Amplicon size range (min, max, average) | % bases over 30X coverage threshold | Amplicons over 30X coverage threshold | % bases over 10X coverage threshold | Amplicons over 10X coverage threshold | Reads | Average read size | Called sequences (Kb) | %  Blast mapping |
| --- | --- | --- | --- | --- | --- | --- | --- | --- | --- | --- | --- |
| 1 | 164 | 50,8 | 121, 569, 313 | 92,9 | 144 | 96,6 | 148 | 128512 | 231 | 28692 | 71 |
| 2 | 103 | 33,9 | 147, 569, 325 | 99,1 | 97 | 99,6 | 98 | 122662 | 229 | 26039 | 77 |
| 3 | 49 | 15,9 | 147, 569, 316 | 98,4 | 45 | 99,6 | 47 | 92188 | 231 | 20420 | 76 |
| 4 | 27 | 8,9 | 121, 569, 308 | 92,7 | 23 | 97,1 | 26 | 30063 | 198 | 5690 | 54 |
| ALL | 343 | 109,5 | 121, 569, 315 | 95,6 | 309 | 98,0 | 319 | 373425 | 222 | 80841 | 73 |

**Table 3**

**Sequence variations identified by GS-FLX pyrosequencing and comparison to standard Sanger sequencing**

| Pool | Confidence | SNP | | | | DELETION - INSERTION | | | | TOTAL | |
| --- | --- | --- | --- | --- | --- | --- | --- | --- | --- | --- | --- |
|  |  | Hetero | | Homo | | Deletions | | Insertions | |  |  |
| Pool-01 |  | Calls | Confirmed (%) | Calls | Confirmed (%) | Calls | Confirmed (%) | Calls | Confirmed (%) | Calls | Confirmed (%) |
|  | TC | 143 | 143 (100%) | 16 | 16 (100%) | 12 | 5 ( 42%) | 2 | 2 (100%) | 173 | 166 ( 96%) |
|  | VC | 21 | 21 (100%) | 1 | 1 (100%) | 11 | 1 ( 9%) | 3 | 3 (100%) | 36 | 26 ( 72%) |
|  | NC | - | - | - | - | 7 | 0 ( 0%) | 5 | 0 ( 0%) | 12 | 0 ( 0%) |
|  | Total | 164 | 164 (100%) | 17 | 17 (100%) | 30 | 6 ( 20%) | 10 | 5 ( 50%) | 221 | 192 ( 87%) |
|  | Sanger |  | 172 ( 95%) |  | 17 (100%) |  | 6 (100%) |  | 5 (100%) |  | 200 ( 96%) |
| Pool-02 |  | Calls | Confirmed (%) | Calls | Confirmed (%) | Calls | Confirmed (%) | Calls | Confirmed (%) | Calls | Confirmed (%) |
|  | TC | 96 | 94 ( 98%) | 10 | 10 (100%) | 12 | 4 ( 33%) | 1 | 1 (100%) | 119 | 109 ( 92%) |
|  | VC | 12 | 11 ( 92%) | 1 | 1 (100%) | 18 | 3 ( 17%) | 2 | 2 (100%) | 33 | 17 ( 52%) |
|  | NC | - | - | - | - | 10 | 0 ( 0%) | 5 | 0 ( 0%) | 15 | 0 ( 0%) |
|  | Total | 108 | 105 ( 97%) | 11 | 11 (100%) | 40 | 7 ( 18%) | 8 | 3 ( 38%) | 167 | 126 ( 75%) |
|  | Sanger |  | 105 (100%) |  | 11 (100%) |  | 7 (100%) |  | 3 (100%) |  | 126 (100%) |
| Pool-03 |  | Calls | Confirmed (%) | Calls | Confirmed (%) | Calls | Confirmed (%) | Calls | Confirmed (%) | Calls | Confirmed (%) |
|  | TC | 36 | 33 ( 92%) | 8 | 8 (100%) | 6 | 4 ( 67%) | 2 | 2 (100%) | 52 | 47 ( 90%) |
|  | VC | 7 | 7 (100%) | - | - | 2 | 2 (100%) | - | - | 9 | 9 (100%) |
|  | NC | 1 | 0 ( 0%) | - | - | 2 | 0 ( 0%) | 6 | 0 ( 0%) | 9 | 0 ( 0%) |
|  | Total | 44 | 40 ( 91%) | 8 | 8 (100%) | 10 | 6 ( 60%) | 8 | 2 ( 25%) | 70 | 56 ( 80%) |
|  | Sanger |  | 43 ( 93%) |  | 8 (100%) |  | 7 ( 86%) |  | 3 ( 67%) |  | 61 ( 92%) |
| Pool-04 |  | Calls | Confirmed (%) | Calls | Confirmed (%) | Calls | Confirmed (%) | Calls | Confirmed (%) | Calls | Confirmed (%) |
|  | TC | 24 | 24 (100%) | 7 | 7 (100%) | 3 | 3 (100%) | 1 | 1 (100%) | 35 | 35 (100%) |
|  | VC | 2 | 2 (100%) | - | - | 5 | 0 ( 0%) | 1 | 1 (100%) | 8 | 3 ( 38%) |
|  | NC | - | - | - | - | 2 | 0 ( 0%) | 3 | 0 ( 0%) | 5 | 0 ( 0%) |
|  | Total | 26 | 26 (100%) | 7 | 7 (100%) | 10 | 3 ( 30%) | 5 | 2 ( 40%) | 48 | 38 ( 79%) |
|  | Sanger |  | 30 ( 87%) |  | 7 (100%) |  | 3 (100%) |  | 2 (100%) |  | 42 ( 90%) |
| All Pools |  | Calls | Confirmed (%) | Calls | Confirmed (%) | Calls | Confirmed (%) | Calls | Confirmed (%) | Calls | Confirmed (%) |
|  | TC | 299 | 294 ( 98%) | 41 | 41 (100%) | 33 | 16 ( 48%) | 6 | 6 (100%) | 379 | 357 ( 94%) |
|  | VC | 42 | 41 ( 98%) | 2 | 2 (100%) | 36 | 6 ( 17%) | 6 | 6 (100%) | 86 | 55 ( 64%) |
|  | NC | 1 | 0 ( 0%) | - | - | 21 | 0 ( 0%) | 19 | 0 ( 0%) | 41 | 0 ( 0%) |
|  | Total | 342 | 335 ( 98%) | 43 | 43 (100%) | 90 | 22 ( 24%) | 31 | 12 ( 39%) | 506 | 412 ( 81%) |
|  | Sanger |  | 350 |  | 43 |  | 23 |  | 13 |  | 429 |

**Table 4**

Analysis of homopolymers.

| Homopolymers for all Pools | | | | | | | | | | |
| --- | --- | --- | --- | --- | --- | --- | --- | --- | --- | --- |
| Length (bp) | A | % miscalls | C | % miscalls | G | % miscalls | T | % miscalls | Total | % miscalls |
| 3 | 897 | 0.11 | 1455 | 0.21 | 1339 | 0 | 1053 | 0 | 4744 | 0.08 |
| 4 | 222 | 0 | 553 | 0.54 | 456 | 0.44 | 320 | 1.25 | 1551 | 0.58 |
| 5 | 79 | 2.53 | 137 | 2.19 | 122 | 4.10 | 99 | 5.05 | 437 | 3.43 |
| 6 | 10 | 30.00 | 24 | 16.67 | 14 | 7.14 | 19 | 10.53 | 67 | 14.93 |
| 7 | 6 | 66.67 | 8 | 62.50 | 7 | 28.57 | 8 | 0 | 29 | 37.93 |
| 8 | 1 | 100.00 | 0 | 0 | 0 | 0 | 3 | 0 | 4 | 25.00 |
| 9 | 3 | 0 | 0 | 0 | 0 | 0 | 5 | 80.00 | 8 | 50.00 |
| All types | 1218 | 0.90 | 2177 | 0.83 | 1938 | 0.52 | 1507 | 1.00 | 6840 | 0.79 |
